# Luminal A and luminal B (HER2 negative) subtypes of breast cancer consist of a mixture of tumors with different genotype

**DOI:** 10.1186/1756-0500-5-376

**Published:** 2012-07-25

**Authors:** Masumi Yanagawa, Kenzo Ikemot, Shigeto Kawauchi, Tomoko Furuya, Shigeru Yamamoto, Masaaki Oka, Atunori Oga, Yukiko Nagashima, Kohsuke Sasaki

**Affiliations:** 1 Departments of Pathology, Yamaguchi University Graduate School of Medicine, Ube-shi, 755-8505, Japan; 2 Departments of Surgery, Yamaguchi University Graduate School of Medicine, Ube-shi, 755-8505, Japan; 3 Division of Breast Disease Surgery, Shimonoseki Kohsei Hospital, Shimonoseki-shi, 750-0061, Japan

## Abstract

**Background:**

The St Gallen International Expert Consensus 2011 has proposed a new classification system for breast cancer. The purpose of this study was to elucidate the relationship between the breast cancer subtypes determined by the new classification system and genomic characteristics.

**Methods:**

Invasive breast cancers (n = 363) were immunohistochemically classified as follows: 111 (30.6%) as luminal A, 95 (26.2%) as luminal B (HER2 negative), 69 (19.0%) as luminal B (HER2 positive), 41 (11.3%) as HER2, and 47 (12.9%) as basal-like subtypes.

**Results:**

The high expression of Ki-67 antigen was detected in 236 tumors; no cases of luminal A subtype showed high expression of the Ki-67 antigen, but more than 85% of tumors of the other subtypes showed high expression. In addition, DNA ploidy and chromosomal instability (CIN) were assessed using imaging cytometry and FISH, respectively. In this series, 336 (92.6%) tumors consisted of 129 diploid/CIN- and 207 aneuploid/CIN + tumors. Diploid/CIN- and aneuploid/CIN+ features were detected in 64.9% and 27.9% of luminal A, 41.1% and 49.5% of luminal B (HER2-), 11.6% and 81.2% of luminal B (HER2+), 4.9% and 90.2% of HER2, and 17.0% and 76.6% of basal-like subtypes, respectively. Unlike the luminal B (HER2+), HER2 and basal-like subtypes, the luminal A and luminal B (HER2-) subtypes were heterogeneous in terms of DNA ploidy and CIN.

**Conclusions:**

It is reasonable to propose that the luminal A and luminal B (HER2-) subtypes should be further divided into two subgroups, diploid/CIN- and aneuploid/CIN+, based on their underlying genomic status.

## Background

Breast cancer is one of the most common malignant tumors in the world, and a large number of patients die of the disease every year. Morphologically and biologically, breast cancer is a heterogeneous disease family comprising a number of subtypes [1]. Although the conventional histological classification system is indispensable for the accurate histological diagnosis of breast cancer, it does not always provide sufficient information to evaluate the biological characteristics of individual tumors and it is not useful for treatment selection. Indeed, it is well known that tumors with the same histological subtypes can have very different biological trajectories. This situation indicates the need for a more reliable classification system, which guides clinical decision-making such as the determination of an optimal therapeutic strategy for individual cancer patients [2,3]. Determining the status of estrogen and progesterone receptors, HER2 amplification and Ki-67 antigen expression is practical and valuable for estimating the patient prognosis and for determination of the treatment strategy [4]. Recently, the St Gallen International Expert Consensus proposed a new intrinsic biological classification system based on the expression of the estrogen receptor (ER), progesterone receptor (PgR), HER2 and Ki-67 [5]. The classification system categorizes invasive breast carcinomas into the following five distinct molecular subtypes; luminal A, luminal B (HER2-), luminal B (HER2+), HER2, and basal-like subtypes, and these subtypes are linked to the therapeutic selection [5]. The classification can be performed in every pathological laboratory where IHC with a simple panel of markers is possible, and this approach is designated as the IHC-based classification [6,7]. The clinical value of this classification system is still ongoing world wide.

The biological characteristics of a tumor are primarily affected by genomic changes. In this context, the nuclear DNA content has been measured to estimate the biological characteristics of solid tumors including breast cancer, and in general, it is accepted that DNA aneuploid cancers represent a poorer prognosis than diploid tumors [8-13]. However, the new classification system has not been evaluated with regard to the status of DNA ploidy and/or CIN. The relationship between the ICH-based subtypes and the status of DNA ploidy and CIN should be elucidated to better understand the differences in the biological characteristics within and between subtypes as a precondition to achieve personalized treatment for breast cancer, and the ICH-based classification system should be evaluated in terms of the status of DNA ploidy and CIN.

In this study, the DNA ploidy and CIN were compared with the subtypes classified by the IHC-based classification system in 363 consecutive invasive breast cancer patients.

## Methods

### Tumor tissue specimens

This study evaluated 363 primary invasive breast cancers. None of the patients had any family history of hereditary breast cancer. The average age of patients was 56.9 years, ranging from 30 to 87 years old. Patients had received neither chemotherapy nor radiation prior to surgery. The Institutional Review Board for Human Use at Yamaguchi University Graduate School of Medicine approved the study protocol, and informed consent for this study was obtained from all patients. Representative parts of the surgically removed tumor tissues were used for touch-smear preparations before fixation. Tumor tissue specimens were fixed in 10% formalin overnight and were subjected to histological examinations including the nuclear grade. An immunohistochemical analysis was performed to classify the breast cancers into molecular subtypes (IHC-based subtypes) [5]. The touch-smear preparations were subjected to fluorescence *in situ* hybridization (FISH) and laser scanning cytometry (LSC) to evaluate the genomic instability status and DNA ploidy, respectively [14].

### Immunohistochemistry (IHC)

The expression status of the ER, PgR, HER2, and Ki-67 antigen was evaluated by an immunohistochemical analysis with antibodies against the ER (1D5, 1:50 dilution, Dako, Denmark), PgR (PR88, no dilution, BioGenex, San Ramon, CA), and Ki-67 antigen (MIB-1, 1:100 dilution, DAKO) in formalin-fixed, paraffin-embedded tissue serial sections as previously described [14]. Prior to immunohistochemical staining, antigen retrieval was performed with microwave heating of tissue sections in a citrate buffer solution at pH 6.0. A cut-off value of 1% for both receptors was used to classify the expression of ER and PgR according to criteria proposed by `The Japanese Society of Breast Cancer’ [15] and others [4,16,17]. When immunostaining was observed in more than 1% of tumor nuclei, the tumor was considered to be positive for the ER or PgR. In brief, breast cancers were classified into five subtypes as follows: luminal A (ER+, PgR+ or PgR-, HER2-, and low Ki-67 index), luminal B (HER2 -) (ER+, PgR+ or PgR-, HER2-, and high Ki-67 index), Luminal B (HER2+) (ER+, PgR+ or PgR-, and HER2+), HER2 (ER-, PgR-, and HER2+), and basal-like (ER-, PgR-, and HER2-).

The expression of Ki-67 antigen was scored for the percentage of tumor cell nuclei with positive immunostaining above the background level by observing at least 1000 tumor cell nuclei (Ki-67 index). In this study, the Ki-67 index was scored as high when 14% or more of the tumor cells were immunostained according to the guidelines of the `St Gallen International expert Consensus’ [5]. All tumors were scored as either high or low according to the Ki-67 index. The immunostained slides were evaluated independently by two of the authors (K. I. and T. F.).

### CIN assessed by FISH

Chromosomal instability (CIN) was examined by FISH using four pericentromeric probes (chromosomes 7, 11, 17, and 18 for D7Z1, D11Z1, D17Z1, and D18Z1, respectively; Abbott Laboratories, Abbott Park, Illinois, IL) on the touch-smear preparations as previously described [18-20], and the presence or absence of CIN was determined according to the degree of variations in the number of FISH spots between nuclei [20-22]. Slides were counterstained with 4’6-diamidino-2-phenylindole (DAPI). CIN was considered to be positive when the fraction of cells with a modal chromosome number was less than 75% for four chromosomes [18-22]. HER2 amplification was tested on the smear preparations for IHC equivocal cases using a PathVysion HER2 DNA Probe Kit (Abbott Laboratories) according to the manufacturer’s instructions as described previously. A tumor was considered to be as positive for HER2 gene amplification when the HER2/CEP 17 ratio was 2.2 or higher.

### Determination of DNA ploidy by LSC

Measurement of the nuclear DNA content by LSC was performed as described previously [20,21]. Briefly, the touch-smear preparations fixed in 70% ethanol were dipped in a propidium iodide solution (25 μg/ml in PBS) containing 0.1% RNase (Sigma-Aldrich Co., St Louis, MO). The DNA content was measured by a laser scanning cytometer (LSC 101; Olympus). Usually, more than 5,000 cells were examined in each sample. A DNA histogram was generated, and the DNA ploidy was determined. DNA ploidy was expressed as the DNA index (DI). A case with 1.0≤ DI <1.2 was classified as a diploid and all others were classified as aneuploid tumors.

### Statistical analysis

The differences in the frequency of marker expression frequency between two groups were determined using the Chi-square test. A difference was considered to be significant for P-values <0.05.

## Results

### IHC-based classification

**Figure 1:**
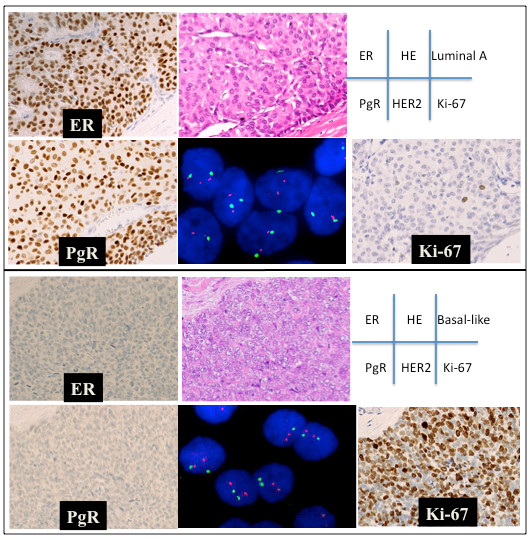
**The results of the immunohistochemical analysis evaluating the expression status of the ER, PgR, and Ki-67 antigens, FISH detecting HER2 amplification, and the histological features in conventional tissue sections stained with hematoxylin-eosin.** Upper rows: A case of invasive ductal carcinoma classified into the luminal A subtype. The ER was expressed in 100% of the tumor cells, and the PgR was expressed in 90% of them. No HER2 amplification was detected (green and red spots indicate chromosome 17 centromeres and HER2). The Ki-67 antigen was expressed in 1% of the cells in this tumor (Ki-67 index = 1%). Lower rows: A medullary carcinoma was classified into the basal-like subtype. The ER and PgR are apparently not expressed in any of the tumor cells. No HER2 amplification was detected. The Ki-67 antigen was expressed in 70% of the tumor cells (Ki-67 index = 70%).

Of the 363 breast cancers, 258 (71.1%) were ER positive and 218 (60.1%) were PgR positive. In this series, 47 (12.9%) tumors were triple-negative (ER and PgR negative expression and no HER2 amplification). The expression of the Ki-67 antigen was deemed positive in 237 (65.3%) tumors (Ki-67+) (Figure 1). HER2 was positive in 110 (30.3%) tumors, which were classified into either the luminal B or HER2 subtype.

### IHC-based subtype classification

According to the recent criteria [5], breast cancers were classified into five IHC- based subtypes as follows: 111 (30.6%) as the luminal A subtype, 95 (26.2%) as the luminal B (HER2-) subtype, 69 (19.0%) as the luminal B (HER2+) subtype, 41 (11.3%) as the HER2 subtype, and 47 (12.9%) for as basal-like (Table 1).

**Table 1:** The status of genomic and cell proliferation markers in the ICH-based subtypes

**Markers**	**LumWalA**	**Luminal B, HER2-**	**Luminal B, HER2+**	**HER2**	**Basal-hke**	**Total**
No. of tumors	111 (30.6%)	95 (26.2%)	69 (19.0%)	41 (11.3%)	47 (12.9%)	363
Age (range)	58.6 (34–84)	54.8 (27–84)	54.7 (30–78)	58,9 (38–87)	56.8 (31–84)	56.7
Ki-67c14%	111 (100%)	0 (0%)	10 (14.5%)	1 (24.3%)	4 (8.5%)	126 (34.7%)
K6714%	0 (0%)	95 (100%)	59 (85.5%)	40 (97.8%)	43 (91.5%)	237 (65.3%)
DiploEd	78 (70.3%)	44 (46.3%)	11 (15.9%)	3 (7.3%)	8 (17.0%)	140 (38.6%)
Aneuploid	33 (29.7%)	51 (53.7%)	58 (84.1%)	38 (92.7%)	39 (83.0%)	223 (61.4%)
CIN-	74 (66.7%)	43 (45.3%)	10 (14.5%)	3 (7.3%)	10 (21.3%)	140 (38.6%)
CIN.	37 (33.3%)	52 (54.7%)	59 (85.5%)	38 (92.7%)	37 (787%)	223 (61.4%)
DipIod!ClN-	72 (64.9)	39 (41.1%)	8 (11.6%)	2 (4.9%)	8 (17.0%)	129 (35.5%)
AneuplodiClN+	31 (27.9%)	47 (49.5%)	56 (81.2%)	372 (90.2%)	36 (76.6%)	207 (57.0%)
Grade 1	53 (47.7%)	15 (15.8%)	6 (8.7%)	0 (0%)	4 (8.5%)	78 (20.4%)
Grade 2	32 (28.8%)	23 (24.2%)	10 (14.5%)	2 (4.9%)	2 (4.3%)	69 (19.0%)
Grade 3	20 (18.0%)	56 (58.9%)	52 (58.9%)	38 (92.7%)	40 (85.1%)	206 (57.3%)
nd	6	1	1	1	1	10

The numbers in the table indicate the number of tumors fitting each item. The numbers in parenthesis are the percentages of tumors fitting each hem. Ki-67+; tumors with a high Ki-67 index, Ki-67-; tumors with a ow Ki-67 index, CIN-; the absence of chromosomal instability, CIN+; the presence of chromosomal instability, Dip/CIN-IKi-67+; tumors with diploidiClN -IKi-67- features, Aneup/CIN÷/Ki-67÷; tumors.

### IHC-based subtypes and Ki-67 antigen expression

**Figure 2:**
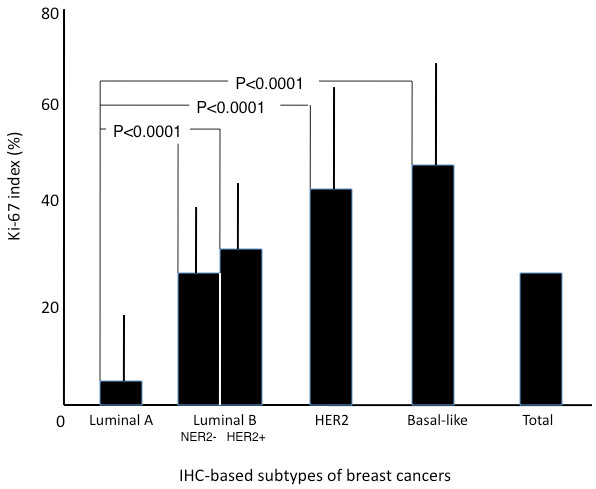
**The relationship between the Ki-67 indices and subtypes.** The high expression (Ki-67 index≧14%) of the Ki-67 antigen was detected in 237 of the 363 breast cancers. The average Ki-67 index was 5.8% (±3.6 standard deviation) for the luminal A subtype, 24.3% (±9.8) for the luminal B (HER2-) subtype, 31.0% (±15.8) for the luminal B (HER2+) subtype, 43.0% (±19.6) for the HER2 subtype, and 46.4% (±23.1) for the basal-like subtype. The difference in the average Ki-67 index was statistically significant between the luminal A and other subtypes (P = 7.366×10^-12^, P = 4.662×10^-12^, P = 4.061×10^-10^, and P = 7.779×10^-4^ between luminal A subtype and luminal B (HER2- & HER2+), HER2, and basal-like subtypes, respectively).

The high expression of Ki-67 antigen was detected in 237 (65.3%) tumors: 0 (0%) of the luminal A tumors, 95 (100%) of the luminal B (HER2-) tumors, 59 (85.5%) of the luminal B (HER2+) tumors, 40 (97.6%) of the HER2 tumors, and 43 (91.5%) of the basal-like tumors (Table 1**)**. The average Ki-67 index was 5.8% (±3.6 standard deviation) for the luminal A subtype, 24.3% (±9.8) for the luminal B (HER2-) subtype, 31.0% (±15.8) for the luminal B (HER2+) subtype, 43.0% (±19.6) for the HER2 subtype, and 46.4% (±23.1) for the basal-like subtype. The average Ki-67 index was significantly different between the luminal A and the other subtypes (P = 7.366×10^-12^, P = 4.662×10^-12^, P = 4.061×10^-10^, and P = 7.779×10^-4^ between the luminal A subtype and luminal B (HER2- & HER2+), HER2, and basal-like subtypes, respectively) (Figure 2).

### DNA ploidy

The DNA indices (DIs) ranged from 1.0 to 3.34 in this series of breast cancers. According to the DIs, the 363 breast cancers were divided into two groups, 144 diploid (1.0 ≤DI <1.2) and 219 aneuploid (DI≥ 1.2) tumors.

### Chromosomal instability (CIN)

In this series, 140 (38.6%) tumors were classified as CIN negative (CIN-), and 223 (61.4%) were classified as CIN positive (CIN+) according to the size of the variant fraction in the chromosome copy number. In this series, 92.8% of the 223 CIN+ tumors were aneuploid, and 94.5% of the 219 aneuploid tumors were CIN+. In contrast, 92.1% of the 140 CIN- tumors were diploid, and 89.6% of the 144 diploid tumors were CIN-.

### DNA ploidy and CIN

Diploid/CIN- features were detected in 129 (35.5%) of breast tumors and aneuploid/CIN- features were detected in 207 (57.0%) tumors, respectively. In this study, 92.6% of the breast cancers were divided into two groups, 129 diploid/CIN- and 207 aneuploid/CIN+ tumors.

### IHC-based subtypes, DNA ploidy and CIN

**Figure 3:**
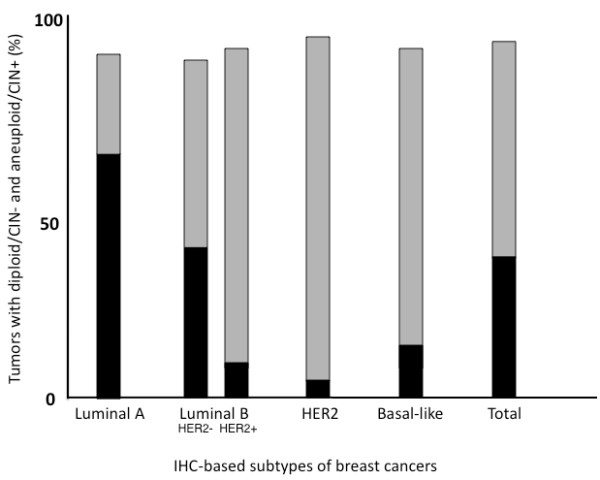
**The relationship of subtypes to the DNA ploidy and CIN.** The diploid/CIN- status was detected in 72 (64.9%) of the 111 luminal A carcinomas, 39 (41.1%) of the 95 luminal B (HER2-) carcinomas, 8 (11.6%) of the 69 lumminal B (HER2+) carcinomas, 2 (4.9%) of the 41 HER2 carcinomas, and 8 (17.0%) of the 26 basal-like carcinomas. In contrast, the aneuploid/CIN + status was detected in 31 (27.9%) of the 111 luminal A, 47 (49.5%) of the luminal B (HER2-), 56 (81.2%) of the 69 luminal B (HER2+), 37 (90.2%) of the 41 HER2, and 36 (86.6%) of the 47 basal-like subtype tumors. The incidence of diploid/CIN- and aneuploid/CIN+ status was different between the luminal A subtype and luminal B (HER2-), luminal B (HER2+), HER2, and basal-like subtypes (p = 0.0006, p = 5E-13, p = 5E10-12, and p = 8E10-9). In addition, the incidence of diploid/CIN- and aneuploid/CIN+ status was statistically different between the luminal B (HER2-) subtype and luminal B (HER2+), HER2, and basal-like subtypes (p = 0.00002, p = 0.000009, and p = 0.002). Black bar; diploid/CIN- tumors, gray bar; aneuploid/CIN+ tumors, white column; others.

The diploid/CIN- status was detected in 72 (64.9%) of the 111 luminal A carcinomas, 39 (41.1%) of the 95 luminal B (HER2-) carcinomas, 8 (11.6%) of the 69 luminal B (HER2+) carcinomas, 2 (4.9%) of the 41 HER2 carcinomas, and 8 (17.0%) of the 26 basal-like carcinomas. The frequency of diploid/CIN- tumors was higher in the luminal A carcinomas than in luminal B (HER2-), luminal B (HER2+), HER2, and basal-like carcinomas (P = 6.315×10^-4^, P = 2.690×10^-12^, P = 5.133×10^-11^, and P = 3.823×10^-8^ between luminal A subtype and luminal B (HER2-), luminal B (HER2+), HER2, and basal-like subtypes, respectively) (Figure 3). In contrast, the aneuploid/CIN+ status was detected in 31 (27.9%) of the 111 luminal A, 47 (49.5%) of the luminal B (HER2-), 56 (81.2%) of the 69 luminal B (HER2+), 37 (90.2%) of 41 HER2, and 36 (76.6%) of the 47 basal-like subtype tumors. The frequency of aneuploid/CIN+ tumors was lower in luminal A subtypes than in luminal B (HER2-), luminal B (HER2+), HER2, and basal-like subtypes (P = 1.482×10^-3^, P = 3.962×10^-12^, P = 6.996×10^-11^, P = 1.525×10^-8^ between luminal A subtype and luminal B (HER2-), HER2 (HER2+), HER2, and basal-like subtypes, respectively) (Figure 3).

In this series, 41.1% and 49.5% of luminal B (HER2-) subtype cases showed diploid/CIN- and aneuploid/CIN+ features , respectively. The proportion of diploid/CIN- and aneuploid/CIN+ in luminal B (HER2-) was similar to that in all tumors, in which the percentage of diploid/CIN- and aneuploid/CIN+ was 35.5% and 57.0%, respectively (Figure 3). The diploid/CIN+ and aneuploid/CIN- classifications were very rare.

### IHC-based subtypes, grade, and DNA ploidy/CIN

**Figure 4:**
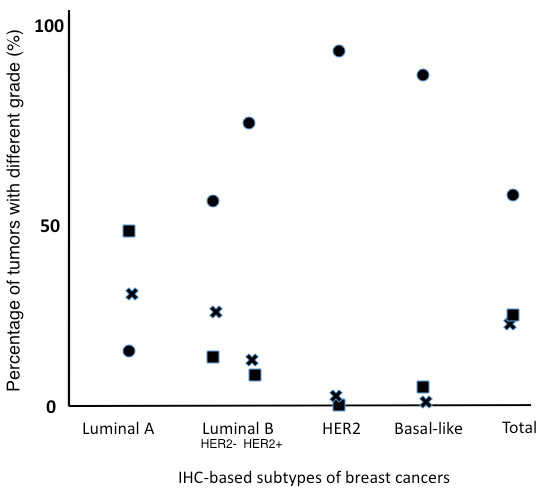
**The relationship between the nuclear grade and subtypes.** Grade 1 tumors were detected in 47.7% of the 111 luminal A subtype tumors, 15.8% of the 95 luminal B (HER2-) subtypes, 8.7% of the 69 luminal B (HER2+) subtypes, 0% of the 41 HER2 subtypes, and 8.5% of the 47 basal-like subtype tumors. Of the grade1 tumors 67.9% were categorized as part of the luminal A subtype. Grade 3 tumors were detected in 18.0% of luminal A tumors, 58.9% of luminal B (HER2-), 75.4% of luminal B (HER2+), 92.7% of HER2, and 85.1% of basal-like tumors. In the HER2 and basal-like subtypes, grade3 tumors were common but grade 1 tumors are rare. In contrast, the luminal A and luminal B (HER2-) subtypes consisted of heterogeneous populations with different grade. Solid square; grade 1 tumors, cross; grade 2 tumors, and solid circle; grade 3 tumors.

This breast cancer series included 78 grade1, 69 grade 2, and 206 grade 3 tumors. Grade 1 tumors were detected in 47.7% of the 111 luminal A subtype tumors, 15.8% of the 95 luminal B (HER2-) subtype tumors, 8.7% of the 69 luminal B (HER2+) subtype tumors, 0% of the 41 HER2 subtype tumors, and 8.5% of the 47 basal-like subtype tumors (Figure 4). Of the grade1 tumors 67.9% were categorized as being the luminal A subtype. Grade 3 tumors were detected in 18.0% of luminal A subtype tumors, 58.9% of luminal B (HER2-), 75.4% of luminal B (HER2+) subtypes, 92.7% of HER2 subtypes, and 85.1% of the basal-like subtype tumors (Figure 4).

**Figure 5:**
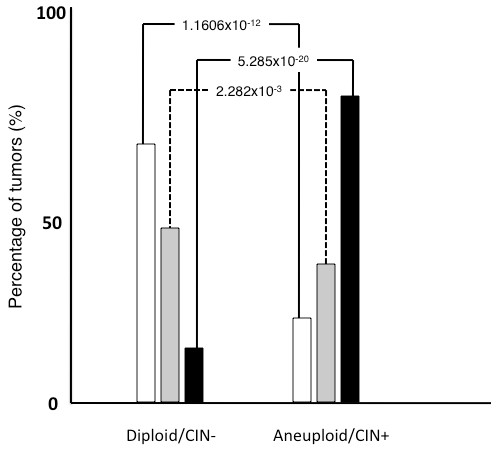
**The differences in the nuclear grades between diploid/CIN- and aneuploid/CIN+ breast cancers.** Grade 1 tumors were more frequently seen in diploid/CIN- carcinomas than in aneuploid/CIN+ carcinomas (p = 1.601×10^-12^). In contrast, grade 3 tumors were much more common in aneuploid/CIN+ tumors than in diploid/CIN- tumors (p = 5.285×10-^20^). The incidence of grade 2 tumors was significantly different between these two types of tumors with different genomic characteristics (P = 2.281×10^-3^). White column; grade 1 tumors, gray column; grade 2 tumors, and black column; grade 2 tumors.

Grade 1 tumors were detected in 54 (41.9%) of the 129 diploid/CIN- cancers and 19 (9.2%) of the 207 aneuploid/CIN+ cancers. In contrast, grade 3 tumors were detected in 35 (27.1%) of the diploid/CIN- cancers and 161 (77.8%) of the aneuploid/CIN+ cancers. Diploid/CIN- and aneuploid/CIN+ features were found in 69.2% and 24.4% of grade 1 tumors, respectively. Diploid/CIN- and aneuploid/CIN+ features were found in 17.0% and 78.2% of grade 3 tumors, respectively (Figure 5).

## Discussion

Recently, a molecular classification system was proposed to categorize breast cancers into subtypes associated with the optimal therapeutic modality, and it has become widely used [5]. In this study, the genomic status together with the cell proliferation activity was compared between IHC-based subtypes.

Although the optimal threshold of the Ki-67 index has been a matter of controversy because of interlaboratory variations, the Ki-67 index has been used to divide breast cancers into tumors with low and high proliferation activity [5,16,23,24]. The expression level of the Ki-67 antigen was generally connected with the IHC-based subtypes [25]. Not surprisingly, all luminal A subtype tumors showed a low Ki-67 index. In contrast, more than 85% of luminal B, HER2 and basal-like subtypes had a high Ki-67 index. It has been suggested that the cell proliferation activity is much lower in luminal A tumors than in other subtypes. The cell proliferation activity as well as other cellular characteristics such as the HER2 expression is a useful marker for categorizing breast cancer, and it is primarily affected by the genomic status.

Determination of DNA ploidy in individual tumors is a simple method to examine crude changes in the genome, but it is very informative. Although aneuploidy and CIN phenotype are different by definition [26,27], almost all aneuploid breast cancers in the present study displayed CIN+ features and vice versa in agreement with previous reports [20,21,27,28]. Smid and colleagues reported that in particular the basal-like subtype showed the CIN+ feature [28]. The majority (93.4%) of breast cancers were divided into either diploid/CIN- or aneuploid/CIN+ tumors. The aneuploid/CIN+ status was the major underlying phenotype in the luminal B (HER2+), HER2, and basal-like subtypes. Approximately 80% of tumors with diploid/CIN- features were of either the luminal A or luminal B (HER2-) subtypes, and a considerable number of aneuploid/CIN+ tumors were also included in these two subtypes. These observations indicate that luminal A and luminal B (HER2-) subtypes can be differentiated from other subtypes based on their underlying genomic changes.

The genomic changes detected by array-based CGH were obviously different between diploid/CIN- and aneuploid/CIN+ breast cancers, as previously reported [28]. DNA ploidy and the CIN status are linked to the cell proliferation activity [29-31]. In addition, aneuploid and CIN+ features are associated with a high proliferation activity and poor prognosis when compared to diploid and CIN- tumors [32]. The DNA ploidy and CIN status were different between luminal B (HER2+), HER2, and basal-like subtypes and the remaining two subtypes. As mentioned above, more than 75% of luminal B (HER2+), HER2 and basal-like subtypes showed aneuploid/CIN+ features . In contrast, 41.1% and 49.5% of the luminal B (HER2-) subtype showed diploid/CIN- and aneuploid/ CIN+ features , respectively. The proportion of diploid/CIN- and aneuploid/CIN+ tumors in the luminal B (HER2-) subtype was similar to that in all tumors. This similarity proves that the luminal B (HER2-) subtype can be further divided into subgroups based on the underlying genomic status. Thus, both luminal A and luminal B (HER2-) subtypes can be divided into two subgroups, diploid/CIN- and aneuploid/CIN+, based on the genomic characteristics of the tumors. Since aneuploid and/or CIN+ tumors show a poorer prognosis [8-13,33,34], the further classification of luminal A and luminal B (HER2-) subtypes may be useful for estimating the prognosis of patients with breast cancer and for clinical decision-making. Further studies are necessary to evaluate the additional classification based on these features. The relationship between CIN and patient prognosis depends on the status of ER expression in breast cancers, paradoxical relationship between CIN and patient prognosis in ER- breast cancers was recently reported [35,36]. Luminal A and luminal B (HER2-) subtypes, which were focused in this study, are ER+ by definition.

The conventional nuclear grading system allows for a rough estimation of the prognosis for breast cancer patients [37]. The nuclear grade of tumor cells can be used as a surrogate marker of the Ki-67 index in the new classification system [5]. In this series, more than 85% of the HER2 and basal-like subtype tumors represented features of grade 3 cancer, whereas the luminal A and luminal B (HER2-) subtypes consisted of mixture of tumors with different grades. The nuclear grade was linked with the DNA ploidy and CIN status. It could be said that the luminal A and luminal B (HER2-) subtypes were heterogeneous both genomically as well as phenotypically. Thus, it is therefore reasonable to propose that the luminal A and luminal B (HER2-) subtypes should be further divided into two subgroups, diploid/CIN- and aneuploid/CIN+, based on their underlying genomic status.

## Conclusions

Unlike the luminal B (HER2+), HER2 and basal-like subtypes, the luminal A and luminal B (HER2-) subtypes were heterogeneous in terms of DNA ploidy and CIN. Thus, it is reasonable to propose that the luminal A and luminal B (HER2-) subtypes should be further divided into two subgroups, diploid/CIN- and aneuploid/CIN+, based on their underlying genomic status.

## Abbreviations

ER, Estrogen receptor; PgR, Progesterone receptor; CIN, Chromosomal instability.

## Competing interests

The authors declare no competing interest.

## Authors’ contributions

MY and KS designed and conducted the study. KI, TF, SK and AO helped with the assays. SY and MO gathered tumor tissue specimens and clinico-pathological data under a code of ethical conduct, MY drafted the manuscript. YN analyzed clinical data. KS conceived of this study, and he participated in its design and coordination. All the authors read and approved the final manuscript.
